# Impairments in Emotion Recognition and Risk-Taking Behavior After Isolated, Cerebellar Stroke

**DOI:** 10.1007/s12311-020-01121-x

**Published:** 2020-02-27

**Authors:** Nils S. van den Berg, Rients B. Huitema, Jacoba M. Spikman, Gert-Jan Luijckx, Edward H. F. de Haan

**Affiliations:** 1 Department of Brain and Cognition, University of Amsterdam, Nieuwe Achtergracht 129B, P.O. Box 15.915, 1001 NK Amsterdam, The Netherlands; 2 Department of Neurology, Unit Neuropsychology, University Medical Center Groningen, University of Groningen, HPC AB60, Hanzeplein 1, P.O. Box 30.001, 9700 RB Groningen, The Netherlands

**Keywords:** Cerebellum, Social cognition, Facial emotion recognition, Risky decision-making, Cerebellar stroke

## Abstract

An increasing amount of research has shown a cerebellar involvement in higher order cognitive functions, including emotional processing and decision-making. However, it has not been investigated whether impairments in facial emotion recognition, which could be a marker of impaired emotional experiences, are related to risky decision-making in these patients. Therefore, we aimed to investigate facial emotion recognition and risky decision-making in these patients as well as to investigate a relationship between these constructs. Thirteen patients with a discrete, isolated, cerebellar lesion as a consequence of a stroke were included in the study. Emotion recognition was assessed with the Facial Expressions of Emotions—Stimuli and Test (FEEST). Risk-taking behavior was assessed with the Action Selection Test (AST). Furthermore, 106 matched healthy controls performed the FEEST and 20 matched healthy controls performed the AST. Compared with healthy controls, patients were significantly worse in the recognition of emotional expressions and they took significantly more risks. In addition, a worse ability to recognize fearful facial expressions was strongly related to an increase in risky decisions in the AST. Therefore, we suggest that tests of emotion recognition should be incorporated into the neuropsychological assessment after cerebellar stroke to boost detection and treatment of these impairments in these patients.

## Introduction

The repercussions of cerebellar damage were originally thought to be restricted to fine motor impairments [1]. Nowadays, it is well known that cerebellar damage affects a broad range of functions, including higher order cognitive functions, which are crucial for everyday life functioning [2]. More specifically, cerebellar damage may result in both cognitive and emotional changes, framed by Schmahmann as the cerebellar cognitive affective syndrome (CCAS) [3]. This can negatively affect adequate decision-making and accurate judgment, which is important in risky or hazardous traffic situations [4].

A range of studies demonstrated cognitive deficits related to the CCAS in patients with cerebellar damage, including executive, spatial, linguistic, and affective deficits [5]. Recently, the focus has shifted to deficits in social cognition and their influence on daily life [6–8]. Social cognition includes those brain functions which enable adequate behavior in social situations [9]. It is a broad construct consisting of different aspects, including the perception of socially important information (such as the recognition of emotional facial expressions), the understanding of others’ behavior (such as creating a theory of mind), and empathic behavior. In a meta-analysis by Van Overwalle et al. [10], in which over 350 fMRI studies were examined, it was shown that the cerebellum plays a modulatory role in different aspects of social cognition, especially in abstract mentalizing, such as thinking about stereotypes or characteristics of others. Hoche et al. [11] found impairments in the ability to understand others’ feelings in patients with different cerebellar disorders. Furthermore, in a recent consensus paper by Adamaszek et al. [12], it was stated that the cerebellum is crucial in different stages of emotion processing, including emotional learning as well as emotional evaluation and subsequent social-behavioral output. To conclude, a growing amount of studies has found a cerebellar involvement in different aspects of social cognition.

A key component of social cognition is the ability to recognize emotional facial expressions [13]. However, this particular aspect of social cognition has hardly been investigated in patients with cerebellar disorders. Some studies included patients with pathological degeneration of the cerebellum, in which other brain structures are often affected as well [14, 15]. Only one study group investigated facial emotion recognition in ischemic stroke patients with isolated, cerebellar lesions, finding clear evidence for a contribution of the cerebellum to emotion recognition, especially to negative emotions [16].

The recognition, understanding, and experience of emotions have been found to be highly interrelated [17]. Hence, worse recognition of emotional facial expressions could be a marker of a decreased ability to feel emotions. A decreased ability to experience emotions can, consecutively, interfere with adequate decision-making behavior [18]. In risky situations, the experience of fear is known to guide decision behavior [19]. Therefore, we propose that impaired fear recognition is related to impaired decision-making in threatening or possibly dangerous situations. This relation has been found in patient groups in which damage to frontal-subcortical circuits underlying social cognition is common, such as traumatic brain injury [20] and Parkinson’s disease [21], but has not been studied yet in patients with cerebellar lesions.

The aim of the current study was twofold. First is to investigate whether stroke patients with discrete and isolated cerebellar lesions showed impairments in emotion recognition and risky decision-making, by comparing them to healthy controls. Second is to analyze whether impairments in emotion recognition in these patients would be related to risky decision-making, which was our expectation.

## Materials and Methods

### Participants

Thirteen patients who had suffered a cerebellar stroke were included. Nine of these participated in a related study assessing stroke patients (FAB4V) and four participated solely in the current study. Both protocols were approved by the medical ethical committee of the University Medical Center (METC-nr 2015.372 and 2018.052). Patients were eligible for inclusion if they had a discrete, isolated, cerebellar lesion on magnetic resonance imaging (MRI) or computed tomography (CT). We included patients from 1 month up to 3.5 years post-stroke. Extra-cerebellar disorders were ruled out by a careful inspection of whole-brain imaging data by an experienced neurologist. Lesions in the supratentorial region were one of the exclusion criteria. Other exclusion criteria included possible dementia (score below cutoff on the Cognitive Screening Test (CST)) [22] and possible depression or anxiety disorder (score above the cutoff on the Hospital Anxiety and Depression Scale (HADS)) [23]. A group of healthy controls (HCs) matched for age, sex, and education was selected from a larger database collected during previous studies. Exclusion criteria for both patients and HCs consisted of substance abuse, history of psychiatric or neurological disorders, or inadequate command of the Dutch language. All participants signed a written informed consent and were treated in accordance with the Declaration of Helsinki.

### Neuropsychological Measures

#### Emotion Recognition

The Ekman 60 Faces Test of the Facial Expressions of Emotions—Stimuli and Test (FEEST) was used as a measure of facial emotion recognition [24]. Sixty faces displaying the basic emotional expressions anger, disgust, fear, happiness, sadness, and surprise, each ten times, are presented in random order to participants, who have to choose which label best describes the expression shown. The six basic emotion scores range from 0 to 10 and the total score ranges from 0 to 60.

#### Risk-Taking Behavior

**Fig. 1:**
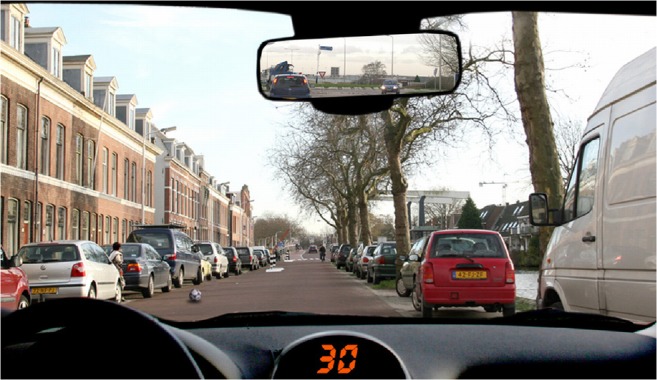
Example of a photograph of the Action Selection Test in the assessment of risk-taking behavior

The Action Selection Test (AST) from the SWOV Institute for Road Safety Research [25] was used to assess risk-taking behavior. Twenty-five photos taken from a car driver’s perspective are displayed on a computer screen. Participants must indicate what they would do if they were in that specific situation: (1) do nothing, (2) release accelerator, or (3) brake. In Fig. 1, an example of a photograph is shown. Here, the correct answer should be “brake,” because of the child running after the ball in front of the car. The responses were rated by experts as very safe, safe, correct, unsafe, or very unsafe [25]. We assigned 0 points to (very) safe or correct responses, one point to unsafe responses, and two points to very unsafe responses. As an indication of risk-taking behavior, we used the sum score of unsafe and very unsafe responses, where a higher score indicates increased risk-taking behavior.

### Imaging

MRI or CT was conducted to verify the lesion location. For five patients, 3D T2-weighted fluid-attenuated inversion recovery (FLAIR) images were obtained as part of the FAB4V-study, using a Siemens Magnetom Prisma 3-T MR scanner (voxel size, 0.9 × 0.9 × 0.9 mm; TI, 1650 ms; TR, 4800 ms; TE, 484 ms; FOV, 280 mm). For five patients, T2-weighted FLAIR images were obtained using a Siemens Magnetom Prisma 3-T MR scanner (voxel size, 0.7 × 0.7 × 4.0 mm; TR, 9000 ms; TE, 81 ms; FOV, 220 mm). Lastly, from three patients, a CT scan was obtained, using a Siemens Definition EDGE, 3rd generation CT scanner (matrix, 512 × 512; voxel size, 0.6 × 0.6 mm; SW, 1.0 mm; FOV, 300 × 300 mm).

### Statistical Analysis

#### Behavioral Analysis

To test for differences in performance on the FEEST and AST between patients and HCs, Mann-Whitney *U* (MWU) tests were performed. Effect sizes were calculated for all between-group comparisons (Hedge’s *g*). To examine the relationship between emotion recognition and risk-taking behavior, Spearman’s rho correlation analyses between all FEEST scores and the AST were performed. In addition, Spearman’s rho correlation analyses were performed between all test measures (FEEST and AST) and lesion size and time since stroke. All analyses were performed with IBM SPSS statistics 23. The alpha level was set at 0.05, but to overcome the multiple testing problem, Bonferroni-Holm corrections were applied to adjust the alpha levels.

#### Imaging Analysis

**Fig. 2:**
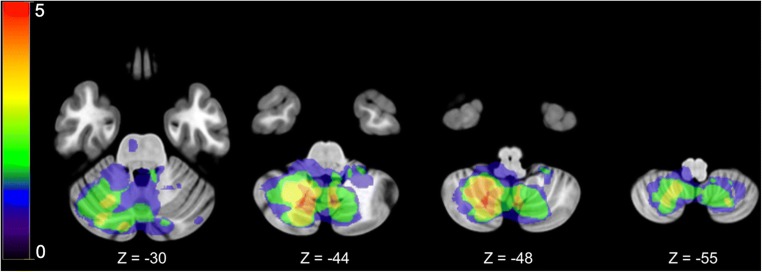
Lesion density plot of the cerebellar lesions on axial slices. Colors indicate the number of overlapping lesions on a range from one (blue) to five (red) overlapping lesions. The left hemisphere is presented on the right

Lesion delineation was performed manually in ITK-SNAP [26]. Subsequently, MRI and CT images and the corresponding lesion maps were normalized using the plug-in clinical toolbox for statistical parametric mapping (SPM) with the stroke control template [27]. MRI templates and CT templates were matched, which allowed us to combine both modalities. A lesion density plot was then created by the normalized lesion maps (Fig. 2).

In addition, atlas-based lesion-symptom mapping (ALSM) was performed to identify regions within the cerebellum related to performance on the FEEST and the AST. In ALSM, the amount of damage in each region is used to predict behavioral test performance. ALSM was performed in NiiStat (https://github.com/neurolabusc/NiiStat), using a probabilistic atlas of the cerebellum [28]. Analyses were restricted to regions in which at least three patients had a lesion. Permutation thresholding (5000 iterations) was used to control for multiple comparisons.

## Results

### Participants

Thirteen patients with discrete, isolated, ischemic lesions in the cerebellum after stroke were included. The time between the cerebellar stroke and inclusion ranged from 1 to 40 months. Demographic data (age, education, and sex) from patients and HCs is presented in Table 1. Patients and HCs were well matched; there were no significant differences between patients and HCs who performed the FEEST with respect to age (*MWU Z* = − 0.07, *p* = 0.95), educational level (*MWU Z* = − 1.60, *p* = 0.11), and sex (*X*^2^ = 1.53, *p* = 0.22). There were also no significant differences between patients and HCs who performed the AST, with respect to age (*MWU Z* = − 0.82, *p* = .41) and sex (*X*^2^ = 0.06, *p* = 0.82). However, patients who performed the AST had a significantly lower educational level than HCs who performed the AST (*MWU Z* = − 2.17, *p* = 0.03).

**Table 1:** Participants’ characteristics

Characteristic	Patients (*n* = 13)	HCs’ FEEST (*n* = 106)	HCs’ AST (*n* = 20)
Age, *M* (*SD*),	61.4 (10.9)	62.0 (8.7)	56.4 (16.4)
Range	43–76	47–80	25–76
Sex, male (%)	11 (84.6%)	72 (67.9%)	16 (80%)
Education, *M* (*SD*)	4.5 (1.1)	5.0 (1.2)	5.4 (0.8)

Education = 7-point scale ranging from 1 (low education) to 7 (university education)

### Emotion Recognition and Risk-Taking Behavior

Table 2 shows the means and standard deviations of HCs and patients on all FEEST scores and the AST, with results of statistical tests for differences. All FEEST scores, except for anger, happiness, and surprise, were significantly lower in patients after the Bonferroni-Holm correction. Furthermore, results for the AST showed that patients took significantly more risks than HCs. The effect sizes (Hedges’ *g*) between patients and HCs were all moderate to large.

**Table 2:** Means (*M*) and standard deviations (*SD*) of the FEEST and the AST for HCs and patients and MWU analyses on differences, with Hedges’ *g* effect sizes between groups

Measure	HC, *M* (*SD*)	Patients, *M* (*SD*)	*Z*	*p*	Hedges’ *g*
FEEST	*n* = 106	*n* = 13			
Anger	7.7 (2.0)	6.4 (2.1)	− 2.1	0.035	0.7
Disgust	7.7 (2.4)	5.2 (3.1)	− 2.9	0.004*	1.0
Fear	5.9 (2.5)	3.9 (1.8)	− 2.7	0.006*	0.8
Happiness	9.8 (0.5)	9.7 (0.6)	− 1.0	0.333	0.2
Sadness	7.2 (1.8)	4.8 (2.2)	− 3.7	0.000*	1.3
Surprise	8.9 (1.1)	7.5 (2.7)	− 1.7	0.099	1.0
Total, range	47.1 (6.0), 28–59	37.2 (7.2), 23–46	−4.3	0.000*	1.6
AST	*n* = 20	*n* = 12^a^			
	4.0 (2.8)	7.7 (4.7)	− 2.7	0.007*	1.0

*FEEST* Ekman 60 Faces Test of the Facial Expressions of Emotions—Stimuli and Test, *AST* Action Selection Test, *HC* healthy control

*Significant at Bonferroni-Holm corrected alpha

^a^The AST is missing for one patient

### Relations Between FEEST and AST

A strong and significant negative correlation was found between the recognition of fear and risk-taking behavior in the AST, indicating that lower fear recognition was related to a higher amount of risky decisions (rho = − 0.85, *p* < 0.001). Correlations between the AST and anger (rho = − 0.01), disgust (rho = 0.05), happiness (rho = 0.03), sadness (rho = − 0.55), surprise (*r* = − 0.12), and the total FEEST score (*r* = − 0.51) were not significant (all *p*s > 0.05). Although not significant, sadness and the total FEEST score were moderately correlated with the AST.

### Lesion Characteristics

Table 3 shows the topography of the cerebellar lesions for each patient. As can be seen in this table and in the lesion density plot (Fig. 2), most lesions were seen in the posterior lobes of the cerebellum, particularly in lobules VI, VIII, and Crus I. In total, twelve patients had lesions in the posterior lobes.

**Table 3:** Characteristics of the cerebellar lesions of all patients, including the hemispheric lateralization, lesion topography, and lesion size (in mm^3^)

Patient	Side	Topography	Lesion size (mm^3^)
1	L	Crus I, Crus II	187
2	B	Left: VI, VIII, IX, Crus I, Crus II; vermis VII, VIII, IX, X; right: VI, VII, VIII, IX, Crus I, Crus II	60,504
3	R	VI, Crus I	1887
4	R	VII, VIII, IX, Crus I, Crus II	12,966
5	B	Left: IV, V, VI; right: Crus I, Crus II	5622
6	R	VI, VII, VIII, IX, X, Crus I, Crus II	22,751
7	L	VIII	719
8	R	VIII, IX	5616
9	L	IX, X, Crus I	3457
10	L	VIII, IX, X	3366
11	L	VII, VIII, IX	9903
12	L	VI, VIII, IX, Crus II	1928
13	B	Left: VII, VIII, IX, Crus II; vermis VII, VIII, IX; right: VII, VIII, IX, Crus I, Crus II	38,543

The ALSM analysis showed a significant association between damage to the left lobule VI and test performance: damage to the left lobule VI predicted increased risk-taking behavior, as measured with the AST (*Z* = 3.34, *p* < 0.001) and a worse ability to perceive the emotional expressions of sadness (*Z* = − 2.61, *p* = 0.0045) and fear (*Z* = − 2.86, *p* = 0.0021), as measured with the FEEST.

### Lesion Size and Time Since Stroke

No significant correlations were found between test performance (all FEEST scores and the AST) and lesion volume (all *p*s > 0.05). Furthermore, no significant correlations between test performance and time since stroke were found (all *p*s > 0.05).

## Discussion

The aim of this study was to investigate whether patients with discrete, isolated, cerebellar lesions after a stroke would show impairments in two relevant aspects of social cognition, i.e., facial emotion recognition and risky decision-making, and whether these impairments would be related to each other. We found that, compared with HCs, patients with cerebellar lesions were significantly worse in the recognition of facial expressions, in particular in the recognition of the negative emotions disgust, fear, and sadness. Furthermore, patients showed significantly more risk-taking behavior in comparison with controls. In line with our hypothesis, worse fear recognition was strongly and significantly related to increased risk-taking behavior in the AST.

This study extends previous research [14, 16] and is the first that has assessed deficits in facial emotion recognition and risk-taking behavior in the subacute and chronic phase in a group of patients with isolated, cerebellar lesions. Most patients in the current study had lesions in the posterior lobes of the cerebellum, including lobule VI and Crus I. An ALSM analysis showed that damage to particularly the left lobule VI was associated with increased risk-taking behavior and with impairments in the ability to perceive sad and fearful faces. These cerebellar areas have previously been found to be related to higher order cognitive functioning and emotional processing [29]. The involvement of the cerebellum in higher order cognitive functions such as social cognition may be explained by the interconnections between the cerebellum and the cerebral cortex, also known as the cerebello-thalamo-cerebro-cortical circuits (CTCCs) [30–32]. Cerebral projections from the cortex are sent to the cerebellum through the pons. The cerebellum sends its output in turn through the thalamus to cerebral areas, including the prefrontal cortex, which is known to underlie social cognition [30, 33]. The cerebellum thus plays a modulatory role in these higher order cognitive functions [4]. Consequently, the cerebellar lesions in our patients may have caused a disturbance in the CTCC, particularly in the connections between the cerebellum and the prefrontal cortex and limbic areas, which could in turn have caused the observed social cognitive problems.

This study furthermore demonstrated for the first time the existence of a relationship between an impaired recognition of fearful facial expressions and risk-taking behavior in patients with isolated, cerebellar lesions. This relation is believed to be mediated by the underlying capability to experience emotions. The recognition and the experience of emotions have been found to be closely related. According to theories of embodied cognition, perceiving an emotional expression includes the embodiment of the corresponding emotion, which aids in the understanding of the emotion [34]. Furthermore, the experience of emotions has been found be crucial in decision-making behavior [18]. Clausi et al. [35] found in a group of patients with cerebellar damage that impaired feelings of regret were related to risky decision-making in a gambling task. Regret is, like fear, a loss-based emotion, which leads to prevention strategies necessary to avoid a bad outcome [36]. According to Zeelenberg et al. [37], each emotion has a specific motivational goal and contributes on its own way to behavior. The emotion of fear has been found to be a warning signal in case of threatening situations, allowing us to choose adaptive and risk-avoiding behavior. Hence, it seems likely that patients in the current study with a diminished fear recognition, suggesting a diminished experience of fear, were not able to use fear to avoid making a risky decision in the AST, a task that highly resembles decision-making behavior in possibly dangerous, everyday life traffic situations. This idea is furthermore supported by findings of Clausi et al. [38], who showed that patients with cerebellar damage have a decreased awareness of their emotional state.

This study is subject to some limitations. First, we assumed that the relationship between emotion recognition and risk-taking behavior was mediated by the underlying ability to experience emotions. It would have been valuable to assess the ability to experience emotions by physiological measures or questionnaires. The merit of using an emotion recognition test is that it is an objective measure which can easily be assessed in patients, even as part of regular clinical care procedures. Furthermore, the sample size was small. However, the strength of our sample is that all patients had isolated lesions in the cerebellum. Lastly, because we did not have T1-weighted MRI images from the majority of patients, we were not able to reliably perform a whole-brain voxel-based morphometry (VBM) analysis to exclude the presence of gray matter degeneration in cortical areas involved in emotion recognition and decision-making, such as the pre-frontal cortex. A reduction of gray matter volume in cerebellar projection areas could have been related to the focal cerebellar damage [39].

## Conclusion

To conclude, this is the first study that investigated emotion recognition and risk-taking behavior in a group of patients with discrete, isolated, cerebellar lesions. We found impairments in emotion recognition, particularly in the recognition of fear, disgust, and sadness, and we found increased risk-taking behavior in patients in comparison with HCs. This study therefore demonstrates a significant contribution of the cerebellum to social cognition. Problems in emotion recognition can have a negative impact on social participation and quality of life after stroke [13]. Furthermore, as we found in the current study, impairments in emotion recognition, particularly fear recognition, can be related to risky decision-making in these patients. We therefore suggest that tests of emotion recognition should be incorporated into the neuropsychological assessment after cerebellar stroke. Possible impairments could in turn be taken into account in setting cognitive rehabilitation goals. This can be very helpful, since these patients seem to be able to compensate for possible cognitive problems [40]. Future studies are needed to extend these results in a broader group of cerebellar patients.

## Abbreviations

ALSM: Atlas-based lesion-symptom mapping
AST: Action Selection Test
CCAS: Cerebellar cognitive affective syndrome
CST: Cognitive Screening Test
CT: Computed tomography
CTCC: Cerebello-thalamo-cerebro-cortical circuit
FEEST: Facial Expressions of Emotions—Stimuli and Test
FLAIR: Fluid-attenuated inversion recovery
HADS: Hospital Anxiety and Depression Scale
HC: Healthy control
MRI: Magnetic resonance imaging
MWU: Mann-Whitney *U*
SPM: Statistical parametric mapping
